# The protective immune response produced in dogs after primary vaccination with the LiESP/QA-21 vaccine (CaniLeish^®^) remains effective against an experimental challenge one year later

**DOI:** 10.1186/1297-9716-45-69

**Published:** 2014-06-25

**Authors:** Virginie Martin, Ioannis Vouldoukis, Javier Moreno, David McGahie, Sylvie Gueguen, Anne-Marie Cuisinier

**Affiliations:** 1 Biological R&D, Virbac, Carros, France; 2 INSERM, UMR S 945, Immunité et Infection, Faculté de Médecine Pitié-Salpêtrière, Université Pierre et Marie Curie, Paris, France; 3 WHO Collaborating Centre for Leishmaniasis, Centro Nacional de Microbiologia, Instituto de Carlos III, Madrid, Spain; 4 Medical Department, Virbac, Carros, France

## Abstract

Control of canine leishmaniasis is an important objective for the benefit of dogs living in or visiting endemic areas and for public health because of the zoonotic nature of this disease. Resistance or susceptibility to developing canine leishmaniasis after exposure to *Leishmania infantum* is primarily determined by the ability of the immune system to develop an appropriate Th1-dominated specific response to the parasite. For this reason there is a need for effective canine vaccines that can decrease the number of dogs developing progressive infections. In this study, we followed the impact of the LiESP/QA-21 canine vaccine (composed of excreted-secreted proteins of *L. infantum* and the QA-21 saponin adjuvant), recently launched commercially in Europe, on selected humoral and cellular immune parameters following an infectious intravenous challenge with *L. infantum* promastigotes administered one year after the primary vaccine course. We also followed parasitological parameters to determine the parasitological status of the challenged dogs. In contrast to controls, vaccinated dogs retained significantly stronger cell-mediated immune responses against the parasite despite a virulent challenge and had significantly lower mean parasite burdens at the end of the study, associated with a lower probability of developing active infections. These results confirm that the immune responses generated by vaccination with LiESP/QA-21 are still effective against an intravenous challenge one year after the primary vaccine course.

## Introduction

Canine leishmaniasis (CanL), a complex disease caused by an inappropriate immune response to infection with *Leishmania* spp, remains a significant problem for the canine population in endemic areas of the world [1]. In Europe, the endemic area is primarily the Mediterranean basin, where the *Leishmania* species responsible is *L. infantum* (*= L. chagasi* of the New World), and transmission is mostly due to the bite of infected sandflies of the *Phlebotomus* (*Larroussius*) subgenus [1,2].

As the primary route of transmission remains the bite of infected sandflies, efforts have been made to reduce the transmission of this disease by use of effective topical repellents and insecticides. Several have trial data demonstrating good short-term efficacy for the reduction of the disease incidence, but some evidence suggests that this may not be sustained over longer periods [3]. Recent work showed that the risk of eventually developing the disease over a prolonged period of time was not directly linked to the level of exposure [4], and further recent work in Portugal confirms that although field use of repellent products helpfully reduces the risk of seroconversion in the dogs at highest risk, this alone is not sufficient to prevent infection [5]. Furthermore, in recent years it has become increasingly clear that rare non-vector transmission of the parasite also occurs [6-8]. The use of repellent products remains a very important tool in the overall management of the epidemiology of CanL, but additional control methods are required [9].

The outcome of infection with *L. infantum* is highly variable in dogs [10]. It is now generally accepted that resistance to developing CanL is primarily dependent on whether the dog develops an appropriate T-helper (Th) 1-dominated cell-mediated immune response against the parasite [11,12]. Therefore, there is a clear need to be able to strengthen a dog’s specific immune response in order to increase the probability that it will correctly manage the infection and remain healthy. In addition, appropriate immune control of parasite replication in turn reduces the reservoir of dogs infectious to naïve sandflies [13]. It is for this reason that several authors have expressed the opinion that an effective vaccine against CanL would be the best control strategy for both canine and human disease [9,14], and there is a growing consensus that an ideal control program for CanL is likely to involve combined use of vaccines with repellent products to maximize the protection of the dog [15].

In recent years two vaccines have been registered in Brazil. Both have a primary course consisting of three injections, followed by annual booster injections. Leishmune^®^ (Zoetis, Brazil) is based on the fucose-mannose ligand of *L. donovani* in association with a saponin adjuvant and demonstrated 76% efficacy against disease or death after natural infection in a field study with evidence of a type 1 immune response being provided by positive intradermal skin test results [16]. The other vaccine available in Brazil is LeishTec^®^ (Hertape Calier, Brazil) which uses the recombinant A2 antigen of *L. donovani* in association with a saponin adjuvant, and demonstrated 43% protection against a culture positive state in an artificial challenge model [17]. A more recent review noted unpublished data suggesting 71% efficacy against a culture positive state in a field study, albeit without the details of the study design [18]. However, until recently no vaccines were available in Europe.

The LiESP/QA-21 vaccine (CaniLeish^®^, Virbac, France), was launched in Europe in 2011. Data demonstrating the rapid onset of an appropriate Th1-dominated cellular immune response after use of the LiESP/QA-21 vaccine, based on specific IFN-γ production in lymphocytes after in vitro stimulation and an increase in the leishmanicidal activity of the macrophages of vaccinated dogs, have been published [19], and it was recently demonstrated that this profile persists for a full year after vaccination [20]. However, as the parasite has a tendency to promote a Th2-dominated profile during infections, it is important to demonstrate that significant challenge with virulent parasites does not reverse the protective immune profile induced by vaccination [21].

This study aimed to assess whether the Th1-dominated immune profile induced by the LiESP/QA-21 vaccine could be sustained in the face of an intravenous experimental challenge (even if this challenge took place beyond the normal time period in which a booster vaccination should have been administered), to assess the ability of this response to effectively reduce the number of dogs developing progressive infections and to assess if vaccination is capable of decreasing the bone marrow parasite load in infected dogs.

## Materials and methods

### Ethics statement

The Virbac Ethical Committee approval confirms that this study was carried out in accordance with the G.R.I.C.E. “Ethical Committee Regulation applied to animal experimentation” guidelines (implemented in France in 2008) under project number 136.01.

### Summary of the study design

This study compared the response of vaccinated and unvaccinated dogs to an intravenous challenge with *L. infantum*. The vaccinated animals had completed the primary vaccination course one year before the challenge. The unvaccinated control group had been maintained in the same conditions during this period. Both groups were then followed for nearly 1 year post-challenge. The details are described below.

### Animals’ characteristics

Twenty conventional Beagle dogs (10 male and 10 female) aged approximately 6 months old (between 5 months and 3 weeks, and 7 months old on the day of the first vaccination), were enrolled into this study. They were evenly assigned to two groups (vaccinated and control) according to their sex and litter of birth. There were 5 males and 5 females per group. They were housed indoor in controlled conditions to avoid any possible risk of natural infection with *L. infantum*.

### Vaccine and vaccination protocol

The LiESP/QA-21 vaccine is authorised in the European Union under the trade name CaniLeish^®^ (Virbac, France). It is composed of purified excreted-secreted proteins of *L. infantum* (LiESP), produced by means of a patented cell-free, serum-free culture system invented by the IRD (Institut de Recherche pour le Développement) [22], and adjuvanted with QA-21, a highly purified fraction of the *Quilaja saponaria* saponin. Dogs in the vaccinated group (*n* = 10) were given one dose of the LiESP/QA-21 vaccine subcutaneously every 21 days for a total of three doses. Vaccinations were administered in the interscapular area. All doses used were formulated at 100 μg ESP and 60 μg QA-21. This represents the minimum accepted antigen level in commercially available doses. Each dose was reconstituted immediately before use in 1 mL 0.9% NaCL solution.

No further booster vaccinations were given during the study. These dogs were therefore “overdue” regarding their booster vaccination requirement at the time of the challenge which took place one year later. Dogs in the control group (*n* = 10) did not receive any vaccination.

### Artificial challenge of dogs

On week 58 (which was 1 year after the last injection of the vaccine), all dogs were challenged intravenously with 10^8.5^ infectious *L. infantum* promastigotes. The challenge innoculum was prepared as follows:

The original isolate of the strain MON1 ITMAP 263 was from the laboratories of the Institut de Recherche pour le Développement (IRD). The strain was passaged on a dog, and after the infection was established the spleen was removed to prepare a master seed of amastigotes. In preparation for the challenge, 2 vials of amastigotes were thawed, and after transformation into promastigotes underwent 4 amplification passages in Schneider complete medium at 25–27 °C for 3 days before being checked for morphology and vivacity to allow the selection of a single culture for the challenge. Morphological assessment confirmed that the parasites were metacyclic promastigotes, as described in Bates and Rogers [23]. These parasites were then washed three times in PBS before being prepared as a suspension of 10^8.5^ parasites/mL in PBS. 1 mL of this solution was administered intravenously in the cephalic vein.

### Analyses and schedule

#### ***Bone marrow sampling to assess parasite load***

On weeks 58, 73, 81, 90, 98 and 105 the dogs were anaesthetized and bone marrow samples were obtained by sternal puncture into citrated tubes. The samples were stored at -70 °C until use, which was always within one week.

On each occasion real-time quantitative PCR (qPCR) was performed to assess the parasite load in the bone marrow. Beginning at week 81, culture was also performed on the samples. These techniques were as described below:

qPCR: After lysis, the DNA of each bone marrow sample was extracted using a silica column (QIAamp DNA mini kit). A Taqman probe was used to amplify and quantify a 200 bp fragment of kinetoplast DNA [24]. Dogs were considered as negative when the titre was inferior to 40 parasites/mL.

Culture isolation: The presence of *Leishmania* parasites was determined by parasite growth on biphasic NNN medium composed of a liquid phase (RPMI medium with 20% FBS) and a solid phase (agar with 10% rabbit blood). The tubes were incubated at 25–27 °C.

Regular microscopic observation was performed to determine the presence of parasites. If no parasites were observed, successive subcultures were realized each week for three weeks. A sample was considered as positive when parasites were observed during the seeding or subculture analysis.

#### ***Clinical follow-up***

Dogs were observed daily to detect any abnormal behavior or clinical signs or any alteration in their general wellbeing. They were also fully assessed clinically once per month, with a specific assessment for signs typically associated with CanL (such as weight loss, splenomegaly, lymphadenopathy, cutaneous lesions, ocular lesions, digestive disturbances, etc.). On weeks 58, 90, 98 and 105 biochemical analysis (Total protein, Albumin, Globulin, Albumin/Globulin ratio) and haematological analysis (White Blood Cell Count, Red Blood Cell Count, Haematocrit, Haemoglobin levels and Platelet Count) were also performed.

#### ***Serology testing of the humoral immune response***

ELISA testing was performed on weeks 58, 73, 90 and 105 to dose the level of IgG1 and IgG2 antibodies to LiESP. Dogs were considered as negative when the titre was inferior to 1/450. On each occasion when ELISA testing was performed, Immunofluorescence testing (IFAT) was also performed on the same samples to dose the level of total anti-*Leishmania* antibodies.

Briefly, the techniques were performed as follows.

##### 

**ELISA** A NUNC Maxisorp plate was coated with 0.1 μg ESP per well in carbonate buffer for 90 min at 35–37 °C. Non-specific sites were blocked with PBS-Tween 0.5%-milk 5% for 90 min at 35–37 °C. Then serial three-fold dilutions of the serum to be tested, from 1/150 to 1/12150, were made in PBS-milk 0.5% buffer and added to the plate. After 60 min of incubation at 35–37 °C any antibodies fixed to the ESP were revealed with a specific peroxydase-conjugated polyclonal anti-IgG1 or anti-IgG2 secondary antibody (Bethyl Laboratories, Montgomery, USA) and ABTS colouration. The titre corresponded to the first dilution with an optical density at 405 nm inferior to 0.4. Dogs were considered as negative when the titre was inferior to 1/450.

##### 

**IFAT** The Fluoleish^®^ kit (BVT, France) was used according to the manufacturer’s instructions using serial dilutions of the serum from the 1/100^th^ to 1/12500^th^. The titre in this test corresponded to the last dilution where at least 50% of the parasites display visible fluorescence.

#### ***Cellular immune response assays***

The three cell-mediated immunity tests, Lymphoblastic Transformation Test (LTT), IFN-γ Enzyme-Linked Immunospot Assay (ELISpot) and Canine Macrophage Leishmanicidal Assay (CMLA) were performed on weeks 58 and 90, and the LTT and ELISpot assays were also performed on week 105. They were performed as summarised below:

##### 

**LTT** This assay is designed to reveal the ability of the specific memory T cells produced as a result of vaccination to proliferate after being exposed to Soluble Leishmania Antigens (SLA). It was performed in a manner similar to that previously described [19,25,26].

Briefly, heparinized blood samples were fractionated by centrifugation over lymphocyte separation medium. PBMCs obtained were incubated at a density of 10^6^ cells/mL for 5 days (37 °C, 5% CO_2_) in presence of either 10 μg/mL ConA, or 10 μg/mL SLA, or with medium alone. The cells were pulsed during the last 24 h with 10 μM 5-bromo-2-deoxyuridine (BrdU), which is incorporated into the DNA of proliferating cells. BrdU incorporation was determined with a specific ELISA system (GE Healthcare, Chalfont St. Giles, UK), using peroxydase-labelled anti-BrdU antibodies which were in turn detected by a substrate reaction using 3,3’5,5’-tetramethylbenzidine. Absorbance values at 450 nm correlate directly to the amount of DNA synthesis and thereby to the number of proliferating cells in culture. The results were expressed as the lymphoproliferation index, which is the ratio of the mean optical density obtained for the SLA stimulated samples compared to the mean optical density obtained for the non-stimulated samples. ConA was used as a positive control and the medium alone was used as a negative control.

##### 

**ELISpot** This assay is designed to determine the proportion of T cells that release IFN-γ after stimulation with SLA in order to quantify the level of stimulation of a specific Th1 polarity immune memory response. It was performed in a manner similar to that previously described [19,27]. Heparinized blood samples were fractionated by centrifugation over lymphocyte separation medium. The PBMCs obtained were incubated at a density of 10^6^ cells/mL for 3 days in multiscreen HTS filter plates (Millipore, Billerica, USA) previously coated with canine IFN-γ capture antibody (R&D System, Minneapolis, USA), in presence of 10 μg/mL ConA, or 10 μg/mL SLA antigens, or with medium alone, in a humidified 37 °C CO2 incubator. The quantity of IFN-γ was revealed with a specific biotinylated antibody and incubation with Streptavidin-AP and the BCIP/NBT Chromogen (R&D System, Minneapolis, USA). The number of specific spots was determined by an automated ELISpot reader. ConA was used as a positive control and the medium alone was used as a negative control. The data presented are the number of spots per 2 × 10^5^ cells after stimulation with SLA minus the equivalent value obtained with the negative control using medium alone.

##### 

**CMLA** This assay is designed to determine the ability of monocyte-derived canine macrophages to kill Leishmania parasites in a co-culture system due to the stimulation of iNOS expression and the resulting production of NO derivatives when the macrophage is exposed to autologous lymphocytes derived from canine PBMC. It was performed in a manner similar to that previously described [19,28-30].

Briefly, monocytes, separated from lymphocytes by adherence, were cultured at a density of 2 × 10^5^ cells per well at 37 °C and 5% CO_2_ for 6 days in complete RPMI 1640 medium containing 25 mM Hepes.

After 6 days of culture, monocyte-derived macrophages were infected with stationary growth phase *L. infantum* (MCAN/82/GR/LEM 497) promastigotes at a ratio of 1:5 for 5 h; then the cells were washed and fresh medium was added for 24 h and this point was considered as time zero. The cells were checked to ensure that greater than 55% were infected. The infected cells (t0) were washed and then in each well 2 × 10^5^ macrophages were incubated alone or in the presence of 10^5^ autologous lymphocytes for 72 h in complete medium containing additionally 10 mM HEPES and 5 × 10^-5^ M 2-mercaptoethanol. After 72 h of co-culture, the lymphocytes were then removed by several gentle washings, the cell free supernatants were conserved for analysis and the macrophages were fixed in order to evaluate the leishmanial killing. One part of the fixed macrophages was stained with Giemsa and the leishmanicidal activity was determined microscopically by counting in triplicate the number of intact parasites per 100 cells in the macrophages co-cultured with the lymphocytes and contrasting this with the number of intact parasites per 100 cells in the macrophages cultured with the medium alone (no T cells). The difference between these results is the percentage inhibition of the parasite index and is expressed as the CMLA index using the following formula: CMLA index = 100 minus (mean number of amastigotes per macrophage multiplied by the percentage of infected cells when co-cultured with lymphocytes)/(mean number of amastigotes per macrophage multiplied by the percentage of infected cells when cultured without lymphocytes) × 100.

The other part of the fixed macrophages was used to evaluate the % of inducible nitric oxide synthase (iNOS) expression by immunolabelling with NOS specific antibodies, as described previously. Briefly, the cells were incubated with rabbit polyclonal antibodies directed against NOS at a dilution of 1:100 in PBS for 1 h at 4 °C, followed by 3 washes in PBS, then the binding of the antibody was revealed by use of a labelled anti-rabbit IgG in an immunofluorescence assay to determine the percentage of iNOS positive macrophages.

The production of NO_2_ (involved in the NO cascade) was determined in the culture supernatants using the modified Griess reference technique. When evaluating this leishmanicidal activity test, a result was considered as successful when the % inhibition of the parasitic index (CMLA) was associated with the activation of the NO pathway and directly correlated with a significant increase of iNOS expression and the production of NO derivatives.

#### ***Glutathione redox balance***

The dosage of oxidized (GSSG) and reduced (GSH) forms of glutathione (redox ratio) was performed on weeks 58, 73, 90 and 105. The red blood cells contained in a whole blood sample were purified and frozen at -70 °C. After their lysis, the supernatant was collected, diluted at 1/30 in 10 nM phosphate buffer and both GSH and GSSG levels were measured by the glutathione reductase enzyme recycling and modified method [31].

Briefly, the dosing of GSH was carried out by adding S-transferase-glutathione with 1-chloro-2.4dinitrobenzene which transforms the GSH into S-(2.4 dinitrophenyl)glutathione [32]. The dosing of GSSG was carried out with glutathione reductase which reduces GSSG to the sulfhydryl form (GSH) in presence of NADPH, H + as co-factor [33].

The sample was then treated with 1-fluoro-2.4-dinitrobenze and neutralised with KOH containing 3-(N-morpholino) propanesulfonic acids. The solutions were then passed through calibrated separation columns. The activity of the samples, both the GSH and GSSG retention time and detection, were calculated using an internal standard curve on a CX4 apparatus (Beckman) after colorimetric detection using a 5100 A Coulochem detector. The follow-up of the simultaneous oxidation state of NADPH,H + was performed by measuring the decrease of its absorbance at 340 nm and the redox state was evaluated on the basis of the GSH/2GSSG (μmoles/g Hb) ratio potential. All chemicals were obtained from Sigma Co. (France).

### Dog status classification

**Figure 1:**
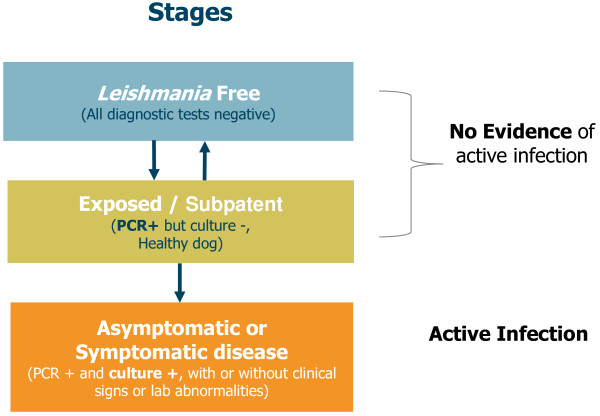
**Classification of the stages of infection used in this study.** Parasitological criteria (quantitative PCR targeting the kinetoplast DNA and culture in biphasic media) were used to classify the status of the dog at the end of the study (week 105) according to the scheme in this figure.

On week 105, the final classification of the dogs was determined using the results of the parasitological tests (see Figure 1). This classification system was adapted from various references in the literature [1,34-36].

Animals which were negative on all tests (PCR negative, culture negative) were classed as “*Leishmania* Free”.

Animals which were PCR positive, but culture negative and otherwise healthy were classed as “subpatent infection”.

Animals which were PCR positive and culture positive, with or without clinical signs, were classed as having “active infection”, which could be symptomatic or asymptomatic.

### Statistical analyses

All statistical tests were performed using the SAS v9.1 software, and for all analyses the significance threshold was set at *p* = 0.05.

Assessments were performed by use of linear mixed-effects models and by non-parametrical means comparison tests as appropriate. The dogs’ status classification on week 105 was compared by treatment group using a Fisher’s exact test.

## Results

### Parasitological status at the completion of the study

On week 105, at the completion of the study, the classification of the dogs in each group was determined to be the following:

Control group – 7 dogs were actively infected, 1 dog was subpatently infected, 2 dogs were free from infection.

**Figure 2:**
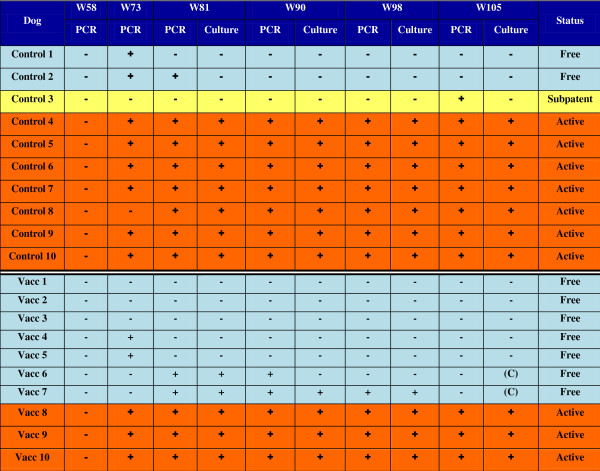
**Parasitological status of control and vaccinated dogs over the course of the study.** This figure presents the individual results of the parasitological tests for each dog, and the resulting status at the end of the study as defined by the scheme in Figure 1. On two occasions the culture medium was contaminated, denoted by (c).

Vaccinated group – 3 dogs were actively infected, 7 dogs were free from infection.These data are represented in Figure 2.

#### ***Quantitative PCR***

**Figure 3:**
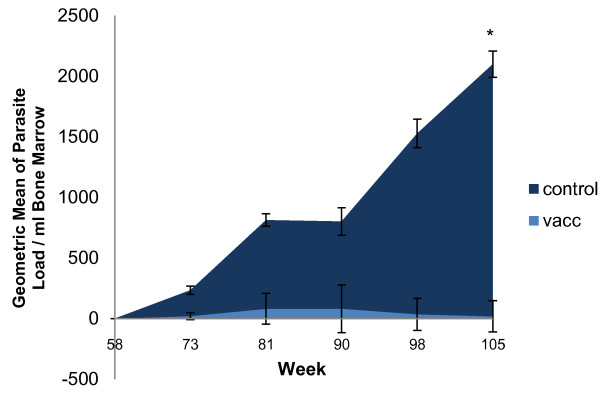
**Geometric means of the bone marrow parasite loads over the course of the study.** The bone marrow parasite loads were assessed by quantitative PCR targeting a 200 bp fragment of the kinetoplast DNA performed on weeks 58 (immediately before the challenge, but one year after the last vaccination), 73, 81, 90, 98 and 105 (equivalent to 47 weeks after the challenge). The number of parasites per ml of bone marrow could thus be calculated, and the results are presented as the geometric mean of the number of parasites per ml bone marrow for each group. Samples were considered as negative if the parasite load was inferior to 40 parasites per ml bone marrow. Error bars represent the SD. *p = 0.026.

Group data for bone marrow parasite loads are presented in Figure 3. Mean values were higher in the control group than in the vaccinated group throughout the challenge period. On week 105, three vaccinated and eight control dogs were PCR positive, with a significant difference between groups (*p* = 0.026).

#### ***Culture***

In the control group, seven dogs were culture positive by week 81, and remained culture positive for the remainder of the study. The other three dogs remained culture negative at each time point.In the vaccinated group, five of the dogs were culture positive on week 81 (the first culture performed after challenge). Three of these (Dogs V8, V9, V10) remained culture positive for the remainder of the study. However one returned to culture negative by week 90 (Dog V6). The other was culture positive until week 98 (Dog V7). On week 105 there was contamination in the culture media for this dog, and so it was not possible to obtain conclusive results. However, at this time point the dog had returned to a PCR negative state, which would not normally be compatible with a positive parasite culture. See also Figure 2 for the details of each dog.

### Clinical signs

Both groups of dogs grew normally during the early period of the study with no differences between them. The mean weight remained stable in both groups for the duration of the challenge period.

No adverse events (local or systemic) were reported after any vaccination.

Three vaccinated dogs presented isolated episodes of mild to moderate hyperthermia during the challenge phase of the study (>39.5 °C) unrelated to the time of the challenge (dog V5 on week 88 and week 100, dog V6 on week 92, and dog V8 on week 105). Two of these dogs (V5 and V6) had been PCR positive (one had also been culture positive) and both then returned to be PCR negative before the hyperthermia episode was recorded. The other (V8) was actively infected at the time. Three control dogs (dogs C1, C2 and C6) also presented isolated episodes of mild to moderate hyperthermia (>39.5 °C), but always during the 8 weeks that followed the challenge. There was no significant difference in mean rectal temperature between the groups throughout the study.

Due to the duration of the challenge phase of this study, it was anticipated that significant clinical signs may not develop in the dogs. However, three dogs in the control group (dogs C3, C4 and C7) developed skin lesions during the second half of the challenge period that were consistent with leishmaniasis. All were infected dogs. One dog in the vaccinated group (V9, the dog with the highest bone marrow parasite load) developed a greasy seborrhea on week 105 at the end of the study. No dog developed severe signs typical of leishmaniasis.

One vaccinated dog (V4) developed a marked popliteal lymph node hypertrophy 3 weeks after challenge, but this resolved over the following 3 weeks. This dog was one of the two to be PCR positive at the first PCR assessment and then negative thereafter. It was assumed therefore to be due to a strong immune response to the massive parasite challenge administered.

### Biochemical and haematological analyses

After challenge, values for white blood cells and platelets were slightly lower than the normal range in some dogs of both groups, but all returned to normal by the end of the study except in dogs C7 and V8 which displayed thrombocytopaenia from weeks 90 and 98 respectively. No abnormal values for red blood cells were detected in any dog.

There was a slight reduction in the mean albumin/globulin ratio in both groups from week 58 to week 98 which then stabilized until the end of the study. Three dogs from each group (V2, V8, V9, C4, C8 and C10) had an albumin/globulin ratio < 0.9 at the end of the study. No dog had hyperproteinaemia at any point.

There were no significant differences between the two groups throughout the duration of the study for any biochemical or haematological parameters.

### Serology testing of the humoral immune response

After challenge, there was a boost of the antibody production in the vaccinated dogs.

#### 

**IFAT** All dogs were IFAT negative at the time of the challenge in both groups.

In the vaccinated group 9 of the 10 dogs had seroconverted on week 73, and the other dog (V9) was IFAT positive on week 90. IFAT titres remained less than 4 times the threshold in dogs that were not actively infected (ranging from 1/200 to 1/500). Only two actively infected (V8 and V10) dogs in the vaccinated group reached titres greater than 1/1000. However one dog (V7) that became temporarily actively infected but then blocked the infection was transiently positive at 1/1000 on week 90 before returning to 1/200 by week 105 after reverting to a PCR negative state.

In the control group, 6 of the 7 actively infected dogs seroconverted with IFAT (on week 90 for 2 dogs and week 105 for the other 4 dogs), with titres ranging from 1/200 to 1/1000. The three dogs that did not progress to active infection (they were never culture positive) and one single dog that was actively infected (C8) did not seroconvert with IFAT. See also Table 1 for details.

**Table 1:** **Anti-*L. infantum *** **IgG titres as assessed by IFAT**

**Group**	**Persistent infection**	**Dog**	**W58**	**W73**	**W90**	**W105**
**Vaccinated**	**No**	**V1**	(-)	1/500	1/200	1/200
		**V2**	(-)	1/200	1/200	1/200
		**V3**	(-)	1/200	1/200	1/200
		**V4**	(-)	1/200	(-)	1/200
		**V5**	(-)	1/200	1/500	1/200
		**V6**	(-)	1/200	1/500	1/500
		**V7**	(-)	1/500	1/1000	1/200
	**Yes**	**V8**	(-)	1/200	1/500	1/2000
		**V9**	(-)	(-)	1/200	1/500
		**V10**	(-)	1/500	1/2000	1/2000
**Control**	**No**	**C1**	(-)	(-)	(-)	(-)
		**C2**	(-)	(-)	(-)	(-)
	**(Subpatent)**	**C3**	(-)	(-)	(-)	(-)
	**Yes**	**C4**	(-)	(-)	(-)	1/500
		**C5**	(-)	(-)	1/1000	1/500
		**C6**	(-)	(-)	1/1000	1/200
		**C7**	(-)	(-)	(-)	1/500
		**C8**	(-)	(-)	(-)	(-)
		**C9**	(-)	(-)	(-)	1/200
		**C10**	(-)	(-)	(-)	1/500

The IFAT titre was assessed using a commercially available kit, and the titre corresponds to the last dilution where at least 50% of the parasites display visible fluorescence. This table presents the individual titres for each dog over the course of the study.

#### 

**ELISA** 7 of the 10 vaccinated dogs retained, or re-developed, IgG1 titres against LiESP after challenge with maximum titres between 1/450 and 1/4050. None of the control dogs developed detectable anti-ESP IgG1 titres.

All vaccinated dogs retained or re-developed anti-ESP IgG2 titres after challenge (range 1/450 to 1/4050). Five of the control dogs also developed anti-ESP IgG2 titres (range 1/450 to 1/1350).

IgG1 and IgG2 anti-ESP titres were not correlated with resistance or susceptibility to the infection.

### Cellular immune response assays

#### 

**Figure 4:**
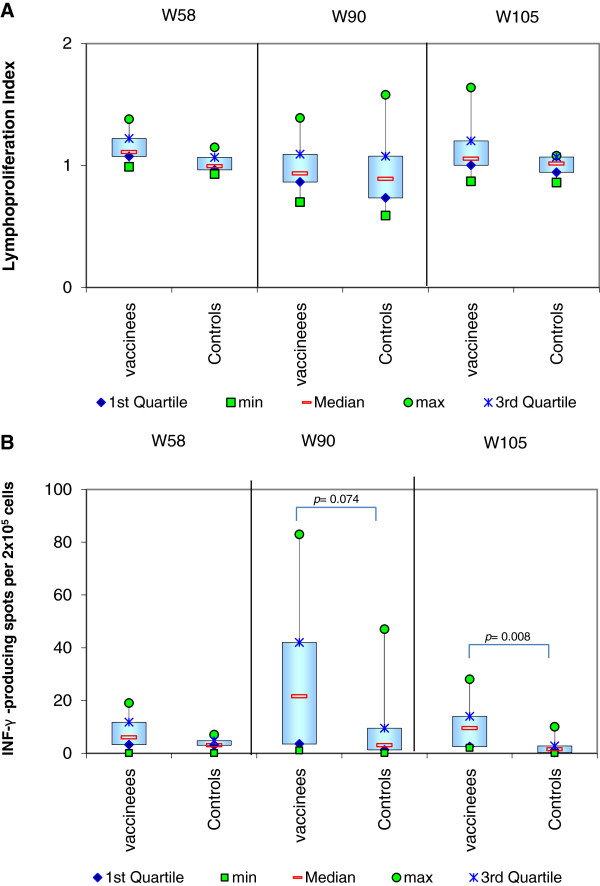
**Lymphoproliferation responses and production of IFN-γ after Soluble *Leishmania *Antigen-specific stimulation. Panel A**: Soluble *Leishmania* Antigen-specific lymphoproliferation. This assay detects the ability of the specific T cells to proliferate after being exposed for 5 days to Soluble *Leishmania* Antigens (SLA). The cells were pulsed during the last 24 h with 10 μM 5-bromo-2-deoxyuridine (BrdU). The lymphoproliferation index is the ratio of the mean optical density obtained for the SLA stimulated samples compared to the mean optical density obtained for the non-stimulated samples using peroxydase-labelled anti-BrdU antibodies. This figure presents the results before challenge on week 58 (W58), which was also one year after the last vaccination, and on weeks 90 and 105. **Panel B**: ELISpot detection of IFN-γ secreting lymphocytes. This assay detects the ability of lymphocytes to secrete IFN-γ after 72 h specific stimulation with Soluble *Leishmania* Antigens (SLA) in coated wells with canine IFN-γ capture antibody. The clones of cells secreting IFN-γ (or spots) were detected using specific biotinylated antibodies and incubation with Streptavidin-AP and the BCIP/NBT Chromogen. The data presented here are the number of spots per 2 × 10^5^ cells after stimulation with SLA minus the equivalent value obtained with the negative control using medium alone. This figure presents the results before challenge on week 58 (W58), which was also one year after the last vaccination, and on weeks 90 and 105.

**LTT** The cells of all animals in both vaccinated and control groups were able to respond effectively to the non-specific positive control stimulation with ConA. A comparison of the result in both groups after SLA stimulation at each time point is shown in Figure 4 panel A. The challenge appeared to result in a non-significant suppression of the mean specific lymphoproliferative ability in both groups on week 90, but this returned to baseline in both groups by week 105.

#### 

**ELISpot** The cells of all animals in both vaccinated and control groups were able to respond effectively to the non-specific positive control stimulation with ConA. A comparison of the result in both groups after SLA stimulation at each time point is shown in Figure 4 panel B. The challenge resulted in a rise in IFN-γ production in both groups by week 90. In the control group this had fallen back to levels below baseline by week 105. In the vaccinated group, the levels also fell again by week 105, but remained higher than at baseline and significantly different to the control group (*p* = 0.0084).

#### 

**CMLA** In the control group, all dogs were below the threshold for all three parameters at week 58, and despite a slight rise at week 90 none of the dogs reached the threshold level (30) for the CMLA index. Three dogs (C1, C9 and C10) achieved levels of iNOS induction and NO2 production slightly above the threshold for these tests on week 90, but in all three cases this was still lower than the lowest results observed in the vaccinated group.

In the vaccinated group, all dogs were above the threshold for all three parameters at week 58 and also at week 90, with a further rise observed at week 90. Only 3 of the vaccinated dogs (V7, V9 and V10) had CMLA index results below 40 on week 90, and all three of these dogs were actively infected at this time point.

**Figure 5:**
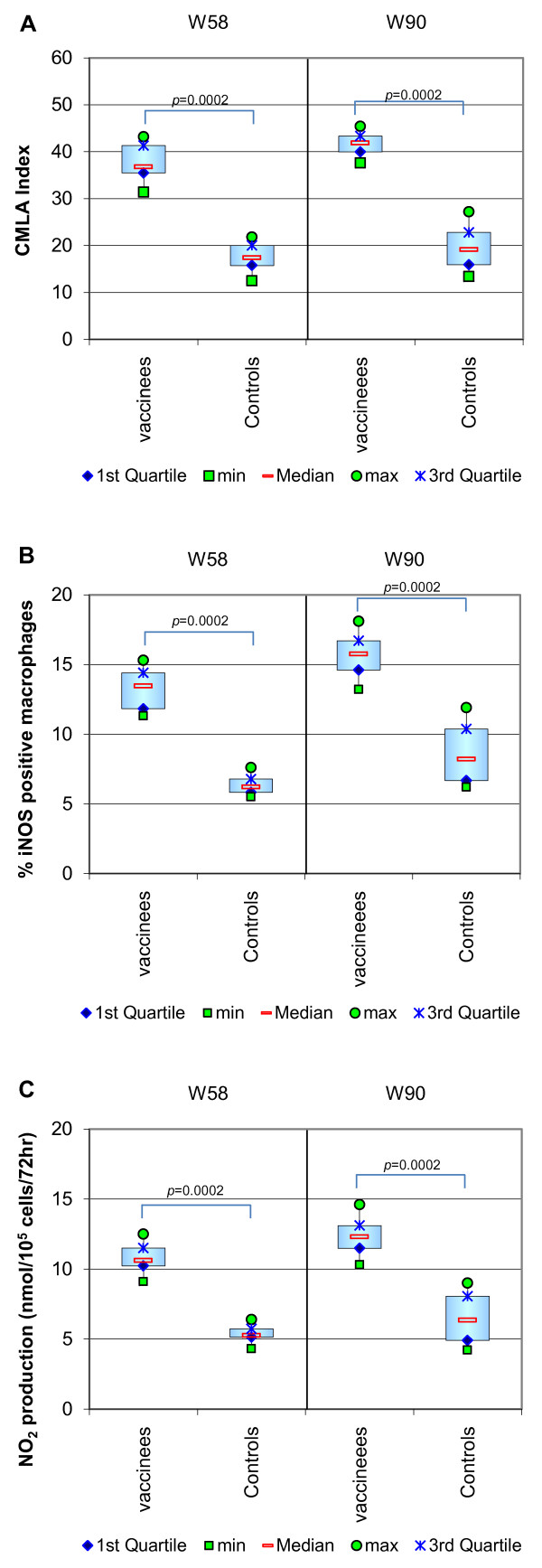
**CMLA assay: inhibition of the macrophage parasitic index, iNOS activity and production of NO derivatives.** Week 58 (W58) was 1 year after primary vaccination, but before the intravenous challenge, and W90 was 32 weeks after the challenge. The macrophages were infected with *L. infantum* promastigotes and incubated alone or in the presence of autologous lymphocytes for 72 h. After this, the lymphocytes were removed, the cell-free supernatants were conserved for analysis and the macrophages were fixed in order to evaluate the leishmanial killing. **Panel A** is a comparison of the ability of the dogs’ macrophages to inhibit parasite multiplication after interaction with autologous lymphocytes. The leishmanicidal activity was determined by counting in triplicate the number of parasites per 100 cells in the macrophages co-cultured with the lymphocytes and contrasting this with the macrophages cultured without the lymphocytes. The difference between these results is the percentage inhibition of the parasite index and is expressed as the CMLA index. **Panel B** is a comparison of the rate of expression of iNOS in the macrophages after 3 days of exposure to autologous lymphocytes. Fixed macrophages were incubated with rabbit polyclonal anti-NOS antibodies. Antibody binding was revealed by labelled anti-rabbit IgG to determine the percentage of iNOS positive macrophages. **Panel C** is a comparison of the rate of production of NO derivatives from the macrophages during co-culture with autologous lymphocytes. NO_2_ was determined in the culture supernatants using the modified Griess technique, providing a correlation with the production of the short-lived NO radical. When the CMLA, iNOS and NO_2_ measurements are consistently increased, this provides evidence of a functional interaction between specific memory lymphocytes and infected macrophages and indicates an increase of NO-mediated parasite killing as a result of vaccination.

The mean values for all three parameters were significantly higher in the vaccinated group compared to the control group both before and after the challenge (*p* = 0.0002 on both occasions for CMLA, iNOS and also NO_2_).A comparison of the results in the 2 groups is shown in Figure 5.

### Redox assay

**Figure 6:**
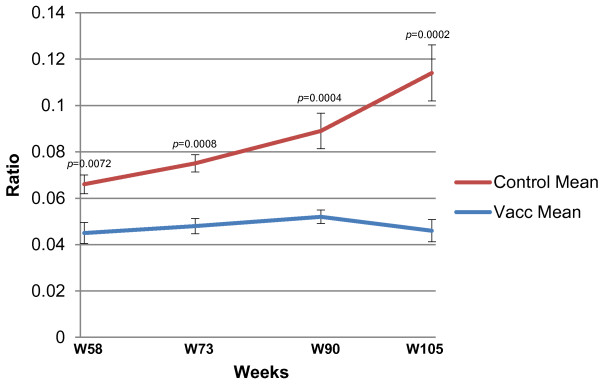
**Redox status of the vaccinated and control groups throughout the course of the challenge period.** The GSH/2GSSG ratio was determined for each dog using purified frozen red blood cells. After their lysis, the supernatant was collected and both GSH and GSSG levels were measured by the glutathione reductase enzyme recycling and modified method. The data are presented as the mean ratio between the reduced (GSH) and oxidised (GSSG) forms in each group with the standard error of the mean. The groups were significantly different at each time point from week 73.

From 15 weeks post challenge (week 73) the oxidised/reduced glutathione ratio began to rise in the control group, but remained stable in the vaccinated group. The groups were different at baseline, but even when taking this into account for all future analyses the difference between groups was highly significant at all points from week 73 (*p* ≤ 0.0008). See also Figure 6.

## Discussion

Experimental studies using artificial virulent challenge are difficult and expensive to perform for canine leishmaniasis. This is due to a combination of factors such as the highly variable outcome of infection, the long incubation period before active infection is established and the loss of parasite virulence after long term in vitro culture that makes it difficult to have large quantities of standardized challenge inoculum [37].

Nevertheless, such studies are an important step in the development of any CanL vaccine. They permit the verification of the in vivo effectiveness of the immune profile induced by vaccination in a relatively small number of dogs over a realistic period of time and provide the necessary level of confidence to proceed to natural challenge studies.

As the dogs in this study were from a single source and were randomized by sex and litter of birth, host variability was reduced to a minimum. In addition, the administration of the same challenge dose from the same batch of parasites to each dog on the same day by the same route reduced challenge variability, and allowed the effects of the vaccine to be studied in highly standardized conditions [38].

Several groups have worked on the development of such artificial challenge models in dogs [37,39,40]. At the time when we started this study, very little detailed data were available and so we had to first validate our own model. However, 2 in vivo challenge studies using intravenous metacyclic promastigote administration had previously been performed with good success using vaccine based on antigen derived from the same patented method of *L. infantum* culture [41,42]. An older study, using only 10^6^ cultured promastigotes of *L. chagasi* appeared to show a protective effect of a different canine vaccine [43]. However a later field study in dogs with the same vaccine could not demonstrate any difference with placebo [44]. It is possible that the use of continuously cultured promastigotes with multiple passage steps, and at lower numbers than we used in our study may have reduced the severity of the challenge sufficiently to allow protection which was not later replicated in the field study. A more recent study used *L. infantum* parasites which were kept virulent by continuous passage in hamsters [45]. However intravenous challenge with 5 × 10^6^ stationary phase promastigotes was still insufficient to induce any clinical signs in dogs, and no conclusions could be drawn on the vaccine efficacy. In a further study using *L. infantum* passaged through hamsters to retain virulence, at 5 × 10^7^ promastigotes per dog [46] the majority of dogs in both groups developed patent clinical signs but the studied vaccine failed to protect the dogs. Finally, in a study using an intravenous challenge with 10^8^ promastigotes of *L. infantum* which went through a small number of culture cycles after being isolated from the spleen of a dog with leishmaniasis [47], clinical signs began to appear in the majority of dogs from week 32 post challenge, and there was a partial protection effect of the studied vaccine.

From these studies, it was clear that the use of parasites obtained from a diseased dog, with a minimum number of passages in culture for the necessary amplification would maximize the likelihood of establishing an active infection in the majority of dogs. They also supported the use of the intravenous route with high parasite doses which had previously been shown to maximize the probability of progressive infections within a reasonable time [37,39,41,42], even if this might carry a risk of underestimating vaccine efficacy [39] while use of lower challenge doses using parasites produced by continuous in vitro culture may overestimate the vaccine efficacy [43,44].

A review published before we established our model [37] suggested that amastigotes may be slightly more efficient at setting up active infections, but that there were other factors that may explain this apparent effect. We therefore chose to use metacyclic promastigotes derived from amastigotes after the minimum number of amplification passages to obtain the required number of parasites. A more recent study using intravenous administration of a very similar number of parasites in the amastigote form, did not appear to lead to a more rapid progression to active infection than that observed in the current study [48].

The production of active infection in the majority of infected animals is in itself is a challenge, as it is clear from the literature that, due to natural resistance, only approximately 1/3 of dogs exposed to the levels of natural challenge commonly found in endemic areas will progress to active infection, and this may take anything from several months to several years to occur [1].

The intravenous route of administration is likely to result in some differences with respect to the establishment of the infection, as it bypasses the local dermal immune environment [39]. Additionally, natural infection is provided by means of sandfly bites, and in that case the parasite is inoculated along with sandfly salivary proteins as well as many internal parasite proteins released from the apoptotic parasites that are also present in the sandfly. These proteins are known to have immune modulating activity, and such effects will not be present with an intravenous challenge [40,49]. For these reasons, artificial challenge studies should be viewed as a final in vivo confirmation of the appropriateness of the immune profile induced by vaccination before proceeding to more extensive natural challenge studies where the true efficacy of the vaccine can be measured. Artificial challenge studies, along with in vitro immune profile studies, can then also support the findings of natural challenge studies performed with the same vaccine [50]. Previous studies had been performed with small batches of a prototype vaccine based on LiESP produced using the same patented culture system but with a different adjuvant. These clearly demonstrated a protective benefit against natural challenge and an immunotherapeutic benefit for dogs with CanL [51,52]. However as the commercially available vaccine does not have the same formulation, it is necessary to assess the immune profile and protection provided with the final formulation.

The choice to perform the challenge without providing the booster was in line with the protocols used in challenge studies for conventional vaccines to confirm the duration of immunity (EMEA Note for guidance: “Duration of Protection Achieved by Veterinary Vaccines” EMEA/CVMP/682/99-Final). However, due to the prolonged incubation time of this disease, the impact of the vaccine was still being assessed nearly a full year after the end of the proposed duration of immunity. In this way a “worst case scenario” was effectively being tested where the immune system was being required to respond over a prolonged period of time based on priming received a full year before the initial challenge.

Regarding the humoral response data, dogs in the control group behaved very much in line with what was observed in previous studies [53,54]. Dogs seroconverted (IFAT or ELISA) only after the dog had become both PCR and culture positive. By contrast, in the vaccinated group seroconversion with IFAT and ELISA was evident at low to moderate levels by the first post-challenge assessment, unrelated to the infection status. It is interesting to note that the three dogs in the vaccinated group that developed active infection were seronegative for anti-ESP IgG2 at the time of challenge, whereas 5 of the 7 resistant dogs were seropositive. Additionally, the dog in the vaccinated group that was slow to re-seroconvert by IFAT (V9), and the dog that was slow to re-seroconvert by ELISA (V8) both developed active infection. In this way, both behaved more like the control dogs with a slower serological response to challenge rather than the rapid memory response that may have been anticipated after vaccination. However it must also be noted that 2 of the resistant dogs in the vaccinated group were seronegative by ELISA at the time of the challenge (V2 and V4), and that both of these dogs developed only threshold IFAT titres during the study and were seronegative by IFAT by week 105, suggesting that the Th1 cell-mediated profile in their immune response was strongly dominant and that the rapid control of the parasite by this means avoided significant stimulation of the humoral response. As a result, great care should be taken when trying to interpret the significance of an individual dog’s serological results in response to a challenge after vaccination. It is not possible to use serological responses to predict the effectiveness of the vaccination in an individual dog.

The cell-mediated immunity parameters provided another set of important information during the challenge period. Although the differences were non-significant, it appeared that the intravenous challenge received suppressed the ability of the lymphocytes to replicate on week 90 in the majority of the dogs, and this is in line with previously reported data [55,56]. Likewise the temporary high peak of IFN-γ production at week 90 as a result of the challenge, most notable in the vaccinated group, was also in line with previously reported data [12,57,58]. In the control group, the return to baseline for the lymphoproliferation index with IFN-γ production at or below baseline levels is likely to be due to specific T-cell exhaustion in the majority of the dogs after a prolonged but ineffective attempt to control the parasite [55]. By contrast, the recovery of *L. infantum*-specific T-cell proliferation in the vaccinated group with the ability produce IFN-γ is probably because in the majority of the dogs the response was effective. It also confirms that the Th1 profile is not lost due to challenge.

The CMLA data are of particular interest. The significant difference observed between the groups in all parameters 1 year after vaccination was retained despite the challenge. In the vaccinated group, the slight rise observed in all parameters indicates that during challenge the capability of the vaccine-induced effector-memory T cells is retained and is further enhanced by exposure to the parasite. In the control group, the fact that the minimum and median values were little changed but that the spread of the results generally increased probably indicates the varied levels of natural resistance which are brought into play subsequent to parasite exposure.

The use of the CMLA test in addition to IFN-γ levels when assessing the immune profile is crucial due to the complex interplay of cytokines including the potential impact of IL-10 as an inhibitory factor and the role of TNF-α as a co-factor of IFN-γ in the control of the macrophage leishmanicidal activity [59]. These cytokine levels could not all be assessed in this study. While it may have been interesting to have looked at the IFN-γ/IL-10 ratio, the positive results obtained in our CMLA assay clearly confirm that the dominant effect of macrophage activators such as IFN-γ is not abolished by a lack of TNF-α or an excess of IL-10.

Even if the parameters used to measure the immune profile confirm that during challenge the vaccine is still able to influence the immune system in the correct way, the primary outcome measure assessed in this study was the parasitological status. The main reason for the use of a CanL vaccine is to reduce the progression to an active infection after exposure to the parasite – the initial infection is introduced mechanically by means of a sandfly bite.

In this study we observed that the two groups behaved quite differently. In agreement with previously published findings, once dogs in the control group developed active infection this was invariably progressive [34], resulting in the continued rise of the mean bone marrow parasite load observed in this group over the course of the study. This can be contrasted with the vaccinated group, where two of the five dogs which had active infection on week 81 (V6 and V7) reverted to a PCR-negative state by the end of the study. This scenario is thought to be highly improbable under normal circumstances in unvaccinated dogs, but confirms that even when challenge levels are extreme enough to overcome natural levels of resistance the vaccine is able to alter the otherwise inevitable progressive nature of a heavy parasite burden and allow the dog to control the infection. In addition, at the end of the study 2 of the 3 vaccinated dogs which remained actively infected were experiencing falling parasite loads, and falling or stable IFAT titres (V8 and V10). This may suggest that they were also beginning to control their parasite burdens. It would have been interesting to follow these dogs for longer, or to observe the impact of a booster injection. It can also be noted that dog C6 experienced a notable drop in the IFAT titre at the end of the study. It is possible that in even rarer cases dogs whose natural resistance is overcome due to the high challenge levels used could eventually control the infection.

High parasite burdens have been associated with higher infectivity to sandflies, and therefore a greater contribution to the local epidemiology of the disease and a greater risk to other dogs and humans [60,61]. Recently, it was demonstrated that when dogs vaccinated with the LiESP/QA-21 vaccine do progress to active infection they are nevertheless still less infective to sandflies than non-vaccinated dogs in the same state [13]. Therefore, if the vaccine can reduce the number of dogs that progress to active infection with a high parasite burden in the first instance, plus provide lower infectivity in those that do progress, these two benefits will be cumulative in reducing transmission. In theory, this would be beneficial for both canine and human health should the vaccine be deployed on a large scale in an area with significant endemicity.

Finally, in this study we also monitored the redox status of the dogs. It has been proposed that oxidative stress plays an important role in the pathogenesis of haemolytic anaemia in visceral leishmaniasis [62], and it has also been suggested that it may play a role in liver and kidney damage due to lipid peroxidation [63]. It has been clearly shown that as dogs develop symptomatic CanL, oxidative stress rises. An oxidative burst provoked by the enzyme NADPH oxidase is typical of the phagocytosis process [64] and large-scale production of reactive oxygen species such as superoxide can therefore indicate an ongoing (but ineffective) cycle of phagocytosis. Experimental evidence suggests that the intracellular redox status regulates various mechanisms of cellular function [65] and that glutathione participates in the induction and maintenance of T-cell dependent immune responses [66]. Glutathione constitutes the first line of the cellular defense mechanism against oxidative injury and the reduced GSH form is considered as an important antioxidant modulator for the immune response during *Leishmania* immunotherapeutics (I. Vouldoukis et al., unpublished results, in preparation). Additionally, it has been demonstrated that the oxidized GSSG form is strongly toxic to the cells. Therefore, a glutathione redox imbalance has been associated with immune system dysregulation, which may then indicate that it not only plays a role in promoting the pathophysiology of the disease, but also that once animals enter this uncontrolled state it is progressively more difficult for their immune systems to control the parasite [67-69]. This may partly explain the fact that after successful chemotherapy of canine leishmaniasis the ability to mount an effective Th1-dominated immune response is once again restored [29]. For this reason, oxidative stress represents an interesting surrogate marker of the level of dysregulation in the immune system during the control of the infection as well as an indicator of damage being done to vital tissues. Thus it is highly desirable to maintain a beneficial cellular redox equilibrium (GSH/2GSSG ratio < 0.1) during the response to challenge. In our study, the highly significant difference in the ratio obtained in the vaccinated dogs compared to control dogs during the response to challenge confirms the ability of the protective immune response induced by vaccination to efficiently control the parasite whilst maintaining an appropriate redox equilibrium.

Taken together, the results presented in this study are highly encouraging and confirm that this vaccine is effective in modulating the immune response to *L. infantum* even after intravenous challenge at the limit of the proposed duration of immunity. Such results must now be confirmed in a larger number of dogs exposed to natural challenge over a longer period of time in order to assess the real efficacy of the vaccine.

### Conclusion

This study has confirmed that even 1 year after the primary vaccination course, and without receiving an annual booster injection, vaccinated dogs are still better able to manage an intravenous parasite challenge. While controlling such a challenge, the immune profile as measured in vitro remains superior in vaccinated dogs, and their redox balance remains normal. Consequently, after vaccination with LiESP/QA-21 the risk of progression to active infection is significantly reduced, as is the bone marrow parasite load, and there is even the possibility to reverse the course of an initial progression to active infection.

## Competing interests

This study was funded by Virbac, which commercialises the vaccine that was the subject of this research. Several of the authors are employees of Virbac. Virbac played a direct role in the study design, data collection and analysis, decision to publish and preparation of the manuscript.

## Authors’ contributions

Conceived and designed the experiments: SG, AMC; Performed the experiments: VM, IV, JM; Analyzed and interpreted the data: DM, AMC; All authors were involved in the drafting and revising of the manuscript, and all authors approved the final version.
